# Comparing Nanomechanical Properties and Membrane Roughness Along the Aging of Human Erythrocytes

**DOI:** 10.3390/mps8040086

**Published:** 2025-08-01

**Authors:** Giovanni Longo, Simone Dinarelli, Federica Collacchi, Marco Girasole

**Affiliations:** Institute for the Structure of Matter, Italian National Research Council (CNR-ISM), Via del Fosso del Cavaliere 100, 00133 Rome, Italy; longo@ism.cnr.it (G.L.); simone.dinarelli@ism.cnr.it (S.D.); federica.collacchi@artov.ism.cnr.it (F.C.)

**Keywords:** erythrocytes, membrane roughness, nanomechanical properties, AFM, cell aging

## Abstract

Erythrocyte (RBC) aging involves significant structural and nanomechanical alterations crucial to their function. This study aims to bridge the gap between analyses based on statistical morphometric parameters, e.g., membrane roughness, and those based on point-dependent nanomechanical properties, e.g., stiffness or Young’s modulus. Using Atomic Force Microscopy, we investigated morphology, membrane roughness, and nanomechanical properties on the very same RBCs under dehydrated (air) and hydrated (physiological buffer) conditions. The cells were studied at different stages of in vitro aging: one, seven, and 12 days. Our results quantitatively show that across dehydration, as well as along the aging pathway, RBCs become progressively more rigid while their membrane roughness decreases, a trend observed in both environments. Notably, the differences between the hydrated and dehydrated states were large in young cells but diminished when erythrocytes aged. Despite these parallel trends, high-resolution mapping on the nanoscale revealed that roughness and Young’s modulus do not correlate, indicating that these parameters are linked to different properties. In conclusion, this work provides a comprehensive protocol for a biophysical description of RBC aging and establishes that the simultaneous measurement of membrane roughness and nanomechanical properties offers a complementary approach, yielding a more complete characterization of cellular properties.

## 1. Introduction

Red Blood Cells (RBCs) are a cell line of great biological and biophysical interest. These cells constitute the universal transporter of gases in the bloodstream (especially O_2_ and CO_2_). Furthermore, being devoid of nucleus and nucleic acids, they present reactivity and biological responses, which are mostly guided by biochemical dynamics. These biosystems have peculiar interconnections between their biological function (including hemoglobin reactivity and metabolism) and their structural architecture, where the morphology and elastic properties of the biosystem are fundamental cellular characteristics.

In addition, erythrocytes represent more than 90% of the cellular components of the blood, so they substantially determine its biophysical and rheological properties, which, in turn, are of heuristic and clinical interest [1,2]. In fact, as blood is a non-Newtonian fluid, understanding its macroscopic behavior requires an accurate description on the microscopic scale of the properties of its elemental components [3,4]. Several experimental and theoretical approaches have been developed that have highlighted the role of the nanomechanical properties of RBCs for these investigations [5,6,7].

A direct consequence of the lack of nucleic acids is that RBCs do not self-replicate. Consequently, they grow older over time, and the fundamental homeostasis of the blood is ensured by a dynamic equilibrium between the production of new cells in the bone marrow and the removal of senescent cells, mostly in the spleen [8,9]. This clarifies the extremely important physiological role played by the erythrocytes’ aging and explains why understanding the chronological steps and the regulation of this complex phenomenon is fundamental in several contexts [10,11]. In particular, the study of the functional and structural alterations that take place during cellular aging is important, not only to better understand the phenomenon, but also because, during aging, the properties of erythrocytes vary progressively according to the characteristics of the environment and to the cellular metabolic and functional alterations. For this reason, the phenomenon has been extensively studied by several groups from different viewpoints [12,13,14,15,16,17,18].

In previous studies, we developed a model system based on a protocol of controlled RBC aging in starvation conditions that proved extremely useful to evaluate physiological and pathological phenomena. As a matter of fact, aging-related alterations can reveal differences in cellular behavior or in the modulation of their properties that could otherwise have easily escaped analysis, even sophisticated analysis, performed in a fixed investigation time [19,20,21].

One quantitative parameter we thoroughly used in previous investigations was the membrane roughness, which describes the nanoscale arrangement of the membrane and reveals untrivial information on the structural integrity of erythrocytes. The measurement of this morphometric parameter was exploited in several case studies of biological and clinical interest, evidencing that the nanoscale arrangement of the membrane does not necessarily correspond to the overall cell’s morphology [19,22,23]. Conversely, this parameter can be extremely useful to elicit new information regarding the structural integrity of the cell and the tightness of the internal connections of the membrane skeleton, which is known to define the morphological properties of these cells.

To achieve these results, the technique of choice is the Atomic Force Microscope (AFM), an investigative tool that allows the quantitative reconstruction, with sub-nanometer resolution, of the morphology of a biological system [24,25,26]. In previous works, we exploited this unique capability to obtain a reliable analysis of RBC’s roughness using a well-defined AFM protocol on a statistically significant and representative set of cells, which were analyzed after controlled dehydration [22,27]. The choice of working on dehydrated cells allows probing a larger number of cells, providing more solid statistical data while causing only minor alterations at a microscopic scale, due to the peculiar characteristics of RBCs (including exceptional skeletal strength due to their structural architecture and the extremely high hemoglobin intracellular concentration). Indeed, even though there is no comprehensive analysis of the effect of dehydration at the nanoscale, accumulated data show that the quantitative differences in the membrane arrangement between healthy and pathological cells, or young and senescent erythrocytes, remained measurable in dehydrated cells, even at the nanoscale.

In recent years, the nanomechanical properties of cells have gained increasing interest in the scientific community due to new evidence on their role in a variety of biological functions (adhesion, migration, mechano-transduction) and their involvement in the occurrence or development of diseases [28,29,30]. This increasing interest has driven attention towards the use of techniques that, like AFM or optical tweezers, can measure or manipulate forces at the nanoscale. In the case of AFM, these measurements are commonly performed through precise and controlled indentation of a surface, which provides a local measurement of the mechanical response of cells [31,32]. In this way, AFM nanoindentation of biological systems is now routinely used to reconstruct full maps of Young’s modulus and cell stiffness, which are the main quantitative parameters used to represent the nanomechanical properties of the cells and are, nowadays, considered of paramount importance in biology [33,34].

Nanomechanical properties in RBCs are mostly determined by their skeleton structure, which has a peculiar composition and an extremely tight connection to the membrane. As a result, the membrane skeleton is capable of reacting to exceptional environmental stress and is responsible for the cell’s elasticity and shape adaptation [35,36,37], which permits the RBCs’ survival during travel in the narrow capillaries. For these reasons, in erythrocytes, nanomechanical properties are extremely interesting parameters and have been studied in various physiological and pathological conditions, such as cancer [30,36,38,39,40], blood-related disorders [40,41], anemia [42], diabetes [38,43], malaria [44,45], obesity [46], neurodegenerative diseases [47,48], or aging diseases [49]. Moreover, in the case of RBCs, the importance of the nanomechanical properties in understanding cellular state overlaps with the role of the morphological characteristics in various contexts, for example, in defining the hemorheological and hemodynamic properties of blood [15,50,51,52,53,54]. The common element in the juxtaposition of nanomechanical and morphological properties lies in the cytoskeleton structure. As a consequence, some studies have focused on investigating the nanomechanical properties of cells (in air or liquid), associated them with the skeleton’s structure, and described the existence and evolution of rearrangements or defects under various environmental stimuli in ghost or reconstituted networks [55,56,57].

Given the interest of the scientific community in these topics, the present work concentrates on coupling nanomechanical properties to a morphometric parameter, the membrane roughness, which can be measured in intact cells. This approach introduces a methodology that promises to close the gap between the biophysical information obtainable with two well-distinct approaches, such as the evaluation of area-dependent statistical morphometric parameters and that of point-dependent nanomechanical properties of the cells.

Besides establishing a proper methodology for the comparison, in the present work, we performed a quantitative evaluation of the effects of dehydration as a physical phenomenon, comparing the nanomechanical and membrane roughness values collected on the very same areas of the very same cells, both in air and in physiological buffer. In this way, we provide for the first time a direct AFM comparison at the single-cell level of the information elicited from quantitative membrane roughness and from nanomechanical investigations. As a further scientific test case, we applied the methodology to the study of the aging pathway of erythrocytes in dehydrated and hydrated states, analyzing the characteristics of young, intermediate, and senescent cells in terms of their nanomechanical and ultra-morphological properties. This investigation allows coupling the monitoring of different cell properties to provide a more complete view of a complex physiological phenomenon, such as the aging of red cells.

## 2. Materials and Methods

### 2.1. Sample Preparation

Blood samples were collected through venipuncture from a healthy donor, after informed consent, and were immediately and carefully transferred into Vacutainers (Becton-Dickinson, Franklin Lakes, NJ, USA) filled with an iso-osmotic buffer containing EDTA (ethylenediaminetetraacetic acid) as an anticoagulant. After gentle stirring, the sample was centrifuged at 3000 rpm for 10 min in a temperature-controlled centrifuge at 4 °C. After the first run of centrifuging, the plasma was removed and stored at 4 °C (to be used later for manual smears), while the white deposit (rich in WBC) on the pellet was discarded. Then, the pellet was resuspended in buffer and washed three more times (10% vol. fraction). Finally, the purified RBCs were maintained in sterile conditions in a buffer (10 mM sodium phosphate, NaCl 140 mM, EDTA 1 mM; pH 7.4) without calcium and glucose and in the presence of phenyl-methyl-sulfonyl-fluoride (PMSF, 1 mM) as a protease inhibitor, which was used to ensure that no proteolytic degradation took place during the aging.

Immediately after the washing and the above-described preparation, an energy synchronization was performed on the cells. Indeed, blood samples contain cells with different aging times and different energetic states. As the aging path is certainly influenced by this factor, an energy reload was provided to the entire population.

The energy reloading procedure (also called rejuvenation) was carried out, according to a slightly modified De Venuto protocol [58], using a rejuvenation solution called IPP. This is an isotonic solution of 10 mM inosine, 10 mM pyruvate, 75 mM sodium phosphate, and 23 mM NaCl, with the pH adjusted to 7.4 using NaOH. Specifically, we depleted the energy of the freshly prepared samples through an incubation for 24 h at 37 °C in a nutrient-free buffer solution, which accelerates their metabolism and consumes most of their residual ATP. After this treatment, the sample was centrifuged for 10 min at 3000 rpm, and the supernatant was substituted with IPP solution in a 1:5 ratio (*v*/*v*) and then incubated again for 3 h at 37 °C. Next, the reloaded RBCs were washed twice and suspended at a final 20% hematocrit in the standard aging buffer. An aliquot of each sample (typically 200 μL) was used immediately before and after the rejuvenation to control the efficiency of the procedure, which typically resulted in a final condition with mM intracellular concentration of ATP (about 7–8 times more than that measured in depleted cells). Measurements of intracellular and extracellular ATP were performed to understand the biological response and the relationship between roughness and ATP during aging. Typical ATP trends were detailed in previous works [19,27] and not presented here, as it is outside the scope of this paper.

The reloaded samples were maintained in the incubation solution at 20 °C for the entire aging time. At selected times, RBC smears were performed manually for morphology analysis through AFM.

After one, seven, and 12 days of aging, sample smears were prepared in duplicate for AFM analysis. They were prepared by adding a 15 μL aliquot of plasma (stabilized at RT) to the same amount of the sample. The use of plasma warrants the best conservation of cell morphology and a homogeneous dispersion on the glass slide during the smear. After gentle mixing, 6 μL of this plasma and sample solution was manually smeared onto a standard poly-L-lysine-coated glass slide (Thermo Scientific, Menzel-Glaser, Waltham, MA, USA) and finally air-dried under laminar air flow.

We previously verified, and now confirmed, that properly performed, and aseptically stored smears are very stable over time and can remain unmodified for years.

All the experiments and sample storage were carried out at room temperature (20 ± 1 °C) and 20% relative humidity.

### 2.2. AFM Setup

For all our experiments, we used a Park NX-12 (Park Systems Inc., Suwon-si, Republic of Korea) Atomic Force Microscope. This microscope was mounted on an Olympus IX inverted optical microscope equipped with a high-resolution camera. This allows for correlative analysis combining optical and AFM imaging of a particular area.

We used Bruker DNP-10 AFM cantilevers (Bruker Co., Billerica, MA, USA), with a nominal tip size of less than 10 nm, choosing the sensor with a nominal elastic constant of 0.12 N/m. Prior to all experiments, the sensors were calibrated using the Park software built-in thermal-noise routines to determine the resonant frequency and the corresponding precise elastic constant of the sensor [59].

All the AFM morphological images in air were collected using the contact mode of the AFM, with a tip-sample interaction below 2 nN.

Once the target cells were determined, we approached the AFM sensor and performed imaging in contact mode. At first, an (approximately) 10 × 10 micron image of an entire cell was collected (with 512 × 512 points), and this was followed by high-resolution images of the cell’s surface, with a typical sampling density of 30–40 points/100 nm.

After imaging, we used the force volume capability of the AFM to collect a grid of force curves from the very same area. The curves were collected using the same tip already employed for the morphological measurements, with a tip speed of 10 μm/s and a maximum applied force of 4 nN. The typical force curve grid was composed of 40 × 40 curves on a 4 × 4 micron area, ensuring a distance between curves of 100 nm. Depending on the morphology of the cell under investigation, in some images and maps, we slightly altered the size and number of force curves. This was repeated on at least 20 cells per sample to ensure statistical significance of the data and repeatability of the measurements.

The same protocols were used for the analyses in liquid. The samples were rehydrated, adding 2 mL of sterilized PBS, and incubated at room temperature for 10 min. Next, we used optical images to identify the cells already imaged in air and repeated the AFM morphology and nanomechanics investigation on the very same cells. Obviously, the collection of force curves on dehydrated or hydrated sample probes the presence of different forces on the sample and may require different strategies for their analysis to ensure an optimal data comparison. For these liquid measurements, we used either contact mode or non-contact mode characterization, employing the same cantilever already used in air, and performed the images with a maximum applied force of 4 nN, while the force curves were performed with a maximum applied force of 6 nN.

### 2.3. Image Analysis and Roughness Calculation

All AFM images were analyzed using the software Gwyddion (version 2.59) [60].

In this study, a major effort was dedicated to the measurement of the plasma membrane roughness.

The determination of the roughness of each cell involved the division of each high-resolution image (typically 3.5 × 3.5 or 4 × 4 μm^2^ with 1200 or 1500 points) of the cell membrane into several square sampling areas of fixed sizes. Indeed, roughness has a strong dependence on the area (as well as on the density of points and on the image resolution), and data collected on areas of different sizes must be analyzed separately. The more extensive roughness evaluation was performed with 300 × 300 nm^2^, 500 × 500 nm^2^, and 1 × 1 μm^2^ sizes; however, sizes of 100 × 100 nm^2^ and 200 × 200 nm^2^ were often considered. The larger (3.5/4 μm) images were treated with simple background subtraction and plane alignment and used for the selection of the sub-images of a smaller size. The processing of each sub-image involved background subtraction, plane alignment, and *x* and *y* axis linearization, while all the residual morphological components were removed by fitting *x* and *y* axes with a 7th-grade polynomial fit. Indeed, the choice of the best polynomial fit was assessed in previous works [27], and the use of a 7th or 8th grade polynomial fit (with differences in the order of few %) resulted as the best compromise between proper subtraction of disturbing low frequency morphological features and preservation of even the tiniest features on the cell membrane. If needed, outliers were removed using the Correct Horizontal Scars and Interpolate small defects features.

After this fitting, the surface roughness (R_rms_) was measured using the formula:

(1) $$R_{rms}=\frac{1}{N-1}\;\times \;\sqrt{\sum_{1}^{N}{\left(X_{i}-X_{m}\right)}^{2}}$$

where *X_i_* is the height value in the *i*-th point, *X_m_* is the average height value, and *N* is the total number of points. The many samplings obtained in different areas of a cell were summarized in an average cell value. When required, an average sample value was obtained by averaging the mean values of the various analyzed cells, and the average sample value was used for comparison between different specimens.

### 2.4. Nanomechanical Properties

The force volume maps were extracted using the Park Systems XEI software (Version 5.2.4 Build 1, Park Systems, Suwon-si, Republic of Korea) to obtain the corresponding batch of single “.txt” force curves. These were analyzed using the freeware software FC_analysis (Version 1.3) [61]. This software allows loading and processing batches of force curves in a semi-automated fashion to obtain, from each force curve, the corresponding values of stiffness (qualitative) and elasticity (Young’s Modulus, quantitative). The particular tip–sample interaction in this case does not allow using the simple Hertz model [62]; therefore, we chose to calculate the Young’s modulus from the force curves using a Bilodeau modified Sneddon model, which, in our experience, is the best model to study the mechanical properties of these cells using pyramidal-shaped tips [63,64]. The software reconstructed the stiffness and Young’s modulus maps in images which were compatible with Gwyddion and other analysis tools.

### 2.5. Statistical Analysis

All the analyses presented in this work have been performed by multiple researchers and compared to ensure unbiased interpretation. The presented data is representative of an overall set of 100 analyzed cells and has been repeated in two independent sample preparations. The statistical analysis of the values was performed using the software Origin Pro (2018, Origin Labs, Northampton, MA, USA). The statistical significance of the roughness and of the mechanical properties were estimated using two-way ANOVA.

## 3. Results and Discussion

The present work has been dedicated to the comparison and integration of the information arising from different AFM-based approaches (morphology vs. nanomechanics) and to the discussion of similarities and divergences in the information they can disclose.

### 3.1. Methodology to Compare Membrane Roughness and Cell Nanomechanics in Air and Buffer

**Figure 1:**
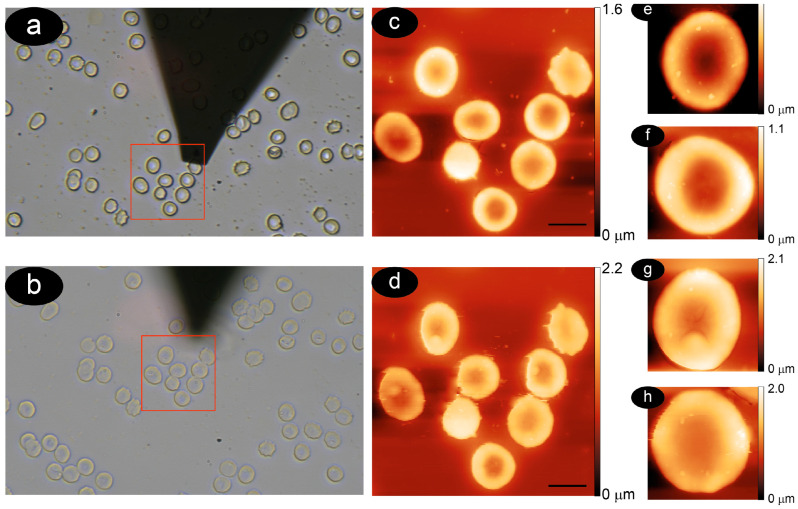
AFM measured in air and in liquid on a specific area of a particular cell (correlative microscopy). In panels (**a**,**b**), the optical images were collected at a 40× magnification; in panels (**c**,**d**), in wide-field AFM topography (the bar indicates five microns); and, in the right panels (**e**–**h**), zoom-in images were collected on two cells. The images were collected on the very same cells in air (top images) and in buffer (bottom images), respectively.

The correlative optical—AFM investigations allowed imaging with increasing magnifications, associated with significant increases in resolution, specific areas of RBCs using the two different techniques. In the same way, the approach allowed repeating the analyses on the exact same area of the sample, and therefore on the exact same cells, even if probed after a few days or in different environmental conditions. This allowed comparing the properties of specific cells in air and in physiological buffer. The approach is described in Figure 1, where we show correlative AFM-optical microscopy of selected cells.

It is worth noting how the overall morphology of the cells collected in air and in liquid does not appear to be influenced by the measurement conditions. Nevertheless, the high-resolution AFM images show the smaller effects caused by hydration, including the removal of vesicles on the cell surface or, sometimes, a slight swelling.

In every sample, we investigated RBCs of different morphology, with emphasis on the most prevalent cell type observed at that time. Indeed, several morphologies are commonly found in the bloodstream.

The most common are the biconcave, but crenated (with oblate edges), spiculed (with thorny or spiky projections on their surface), or spherical cells can be found as well.

**Figure 2:**
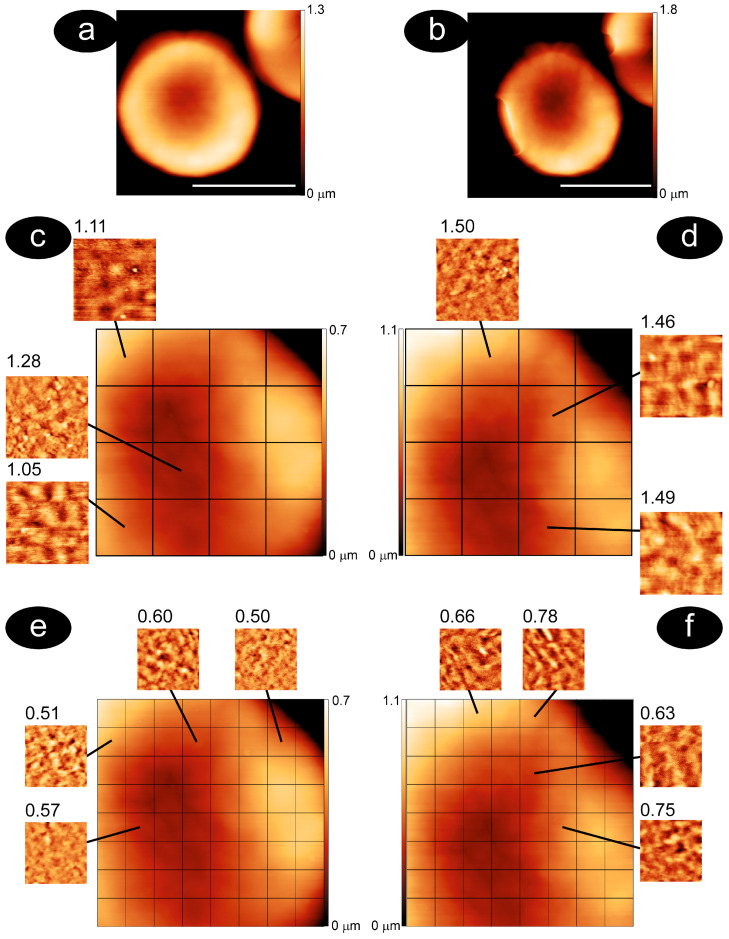
Procedure for the determination of the surface roughness of RBCs. The panels (**a**,**b**) show AFM images of erythrocytes in air and in buffer, respectively (the white bars indicate five microns). The middle and bottom panels show the corresponding zoom-in (4 × 4 μm^2^) on the cell membrane and the division of the membrane area into 1 × 1 μm^2^ (panels (**c**,**d**)) or 500 × 500 nm^2^ squares (panels (**e**,**f**)). The insets show the membrane in the specific squares and the corresponding roughness values.

As aging increases, the size and the original biconcave shape diverge, and the other morphological types become more common [27,65,66]. For instance, at T12, flat or spiculed cells are quite common, while at T7, crenated and spiculed RBCs can be observed. These alterations are inherently part of the aging process, and this morphological evolution is clearly reflected in our data. The procedure employed to determine the roughness of the cell membrane is described in detail in Figure 2. Namely, the surface of a single cell was imaged using the AFM at high resolution in air (panels a, c, e) and buffer (panels b, d, f). The collected image was divided into sub-images with different sampling areas: 1 × 1 µm^2^ and 500 × 500 nm^2^ (the mapping was also performed almost systematically on areas of 300 × 300 nm^2^, while analyses on smaller areas were carried out when necessary). For each sub-image, the roughness value was calculated. In this way, through nanoscale mapping, we simultaneously obtained a visual description of the surface arrangement and a concise mathematical description of it.

By comparing the same areas in dehydrated and hydrated conditions, we were able to highlight small membrane rearrangements, which usually correspond to a variation in the measured roughness values. Remarkably, in almost all measured cells, a significant increase in membrane roughness was observed when moving from dehydrated to properly hydrated cells, especially at small aging times, and this appears to be independent of the overall cell morphology. As an example, in the case of Figure 2, the average roughness calculated from all the sub-images increased when passing from air to buffer: from 1.44 to 2.06 on areas of 1 × 1 µm^2^ and from 0.68 to 0.94 at 500 × 500 nm^2^.

**Figure 3:**
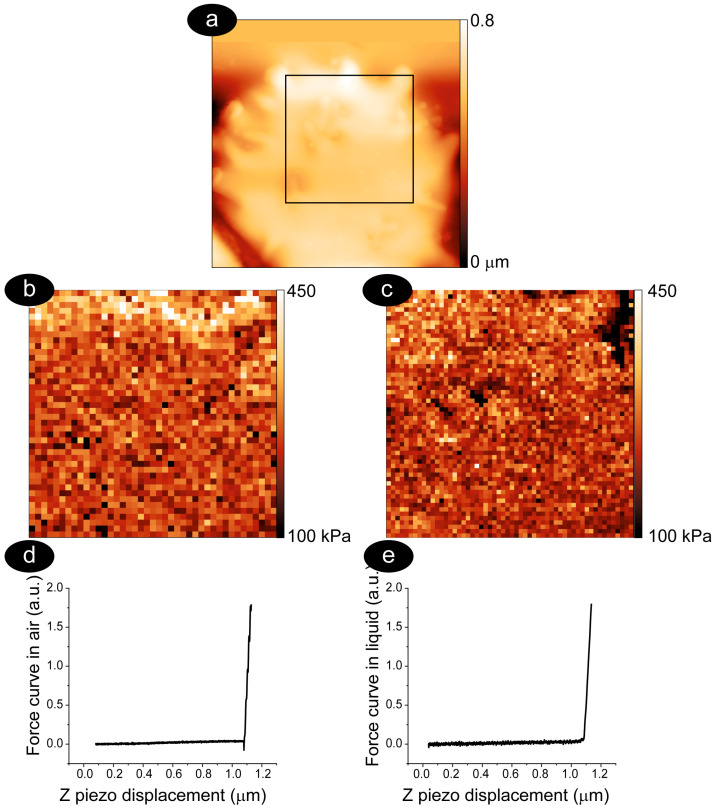
Maps of Young’s modulus measured on the very same cell in the dehydrated and hydrated states. The cell topography is shown as a 6.5 × 6.5 μm^2^ image in panel (**a**), while the force curve maps are reported in panels (**b**) (dehydrated state) and (**c**) (hydrated state). The size of the sub-images is 3.3 × 3.3 μm^2^, and we chose a spiculed RBC, which is rather common at long aging times. Two randomly chosen force curves, one for each map, have been reported (panels (**d**,**e**)) to evidence intrinsic differences due to the different environmental conditions.

The protocol we have described here for the measurement of roughness allows a quantitative analysis of the cell ultra-morphology, enabling a comparison with the nanomechanical properties. Measuring the nanomechanical properties of RBCs involves repeated vertical displacements of the AFM sensor to produce force curves. An example is shown in Figure 3, which reports an image of cell topography (panel a) and a map of its nanomechanical properties determined by collecting a tight grid of force curves. The resulting images, shown in panels b and c, represent maps (or force volume images) of nanomechanical properties (specifically Young’s modulus) collected on the very same cell in air and liquid [67,68].

It should be noted that the Young’s modulus values calculated on biological systems are very much dependent on the tip, the sample, the interaction, the instrument parameters, and, chiefly, on the chosen model used to study the force curves [69,70,71]. This means that different research may show different nanomechanical values for RBCs [47,48,57,72]. Here, the main point to remark is that there must be consistency in measurements and analyses, ensuring that tip properties and indentation procedures are always the same throughout the experiments. This ensures that the morphological and mechanical differences are significant from a physical point of view.

The comparison of the collected maps can measure quantitative differences between samples, but it can also provide interesting information on the process under study, even when no significant differences in the quantitative measured values were observed. Indeed, in the case of the spiculed cell reported in Figure 3, the spatial characteristics of the reconstructed images can be locally different. For instance, the spicules occupying the upper part of the images appear harder in the dehydrated cell than in the corresponding hydrated cases. Other qualitative differences can be seen in other parts of the images, such as in the proximity of morphological structures present in the center and lower parts of the image, which were better evidenced and more defined in the hydrated case.

### 3.2. The RBCs Aging

The scientific problem we used as a test case was the analysis, from the morphological and nanomechanical point of view, of the aging path of human erythrocytes. In particular, we investigated three steps of the phenomenon: an initial phase with young cells rich in ATP (T1), a late phase (T12) with senescent cells that lack ATP, and an intermediate phase (T7) where the biochemical and morphological properties of cells are rapidly changing. Underlining the ATP content is important because previous studies demonstrate how this molecule is a major determinant of the morphological and metabolic evolution that the cells undergo [21,27,73].

**Figure 4:**
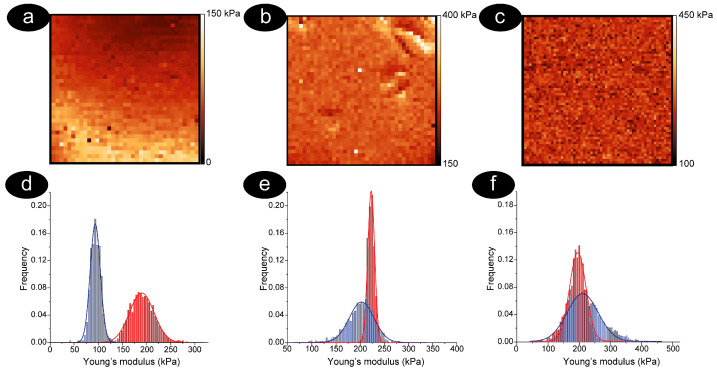
Maps of force curves (panels (**a**–**c**), 3 × 3 μm^2^ images) and relative histograms (panels (**d**–**f**)) reporting the distribution of Young’s modulus measured on a typical biconcave cell at T1 (panel (**a**,**d**)); on a spiculed cell at T7 (panel (**b**,**e**)) and on a flat cell at T12 (panel (**c**,**f**)). For convenience, the reported maps (panels (**a**–**c**)) show cells imaged in air, but the histograms of the Young’s modulus shown in panels (**d**–**f**) refer both to the measurements performed in air (red histogram) and in physiological buffer (blue histogram). The evolution of the histograms clearly describes the overall stiffening dynamics that take place along the aging, and evidence that most of the effect occurs in the first days of aging.

The evolution of the nanomechanical properties of RBCs during aging is described in Figure 4, where maps of typical cells analyzed after one, seven, and 12 days of aging are reported. Young’s modulus maps with sufficient resolution allow understanding the overall morphology of the cells, and, in our case, we have chosen cells with morphologies typical of the aging time considered. This is biconcave in young cells (T1), spiculed cells at T7, and flat with a membrane defect in the case of senescent cells (T12).

The results shown in Figure 4 are discussed for specific cells, but, in fact, they are representative of the typical behavior of the samples at the investigated aging times.

The first thing that can be determined from the data is that the cells were always softer in buffer than in air. In more detail, the differences between the distributions of the nanomechanical properties measured in buffer and air were larger in young cells, smaller at intermediate aging, and almost disappeared in senescent RBCs. Moreover, the quantitative Young’s modulus values, measured in dehydrated cells, change very little over time, while the variations were much more pronounced during the aging of hydrated cells. This evidence shows that in dried cells, already stiffened by dehydration, only small additional changes can be measured during aging. On the contrary, a significant variation of elasticity takes place in the physiological buffer, indicating that aging produces major alterations of this cellular property. This is also expected to occur in vivo.

**Figure 5:**
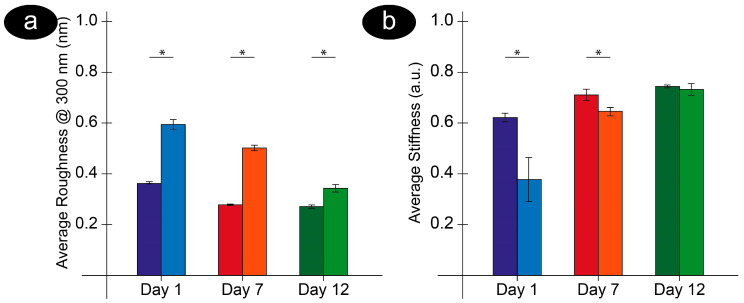
Panel (**a**): trend of average roughness measured at different aging times in air (darker colors) and buffer (lighter colors). The reported roughness was calculated in areas of 300 × 300 nm^2^, but a similar trend was observed in areas of 500 × 500 nm^2^. Panel (**b**): average stiffness calculated on the very same cells, in air (darker colors) and buffer (lighter colors). Clear effects of hydration/dehydration, as well as effects of aging, are evidenced by both parameters. The * indicates the significance of the differences in the values obtained through a two-way ANOVA.

To compare all the measured data, in Figure 5, we report the values of average roughness and average stiffness, measured in air and liquid, on the entire populations of analyzed cells at different aging times. In particular, the trend of the average membrane roughness, measured for an area of 300 × 300 nm^2^ (Figure 5a) after one, seven, and 12 days of aging, shows some interesting characteristics. In agreement with what was observed in previous studies conducted in air, the roughness of the samples decreases continuously with increasing aging. Moreover, dehydration was always associated with a decrease in roughness, and this can be explained by an overall compression and rearrangement of the cell surface that certainly takes place during water evaporation.

Furthermore, comparing the roughness values in liquid and air at increasing times, the data show that the increase in roughness due to hydration significantly reduces along aging, an effect that can be attributed to a general stiffening of the biological structures coupled with a progressive dehydration that takes place during aging. This latter effect, indeed, is intrinsic to the aging phenomenon as, during senescence, the RBCs lose a significant part of their membrane and cellular content.

It is interesting to note that the collected data underlines how the properties of the cells vary during aging, including their capacity for hydration/dehydration. Remarkably, the roughness trend can be directly linked to the average stiffness data measured in air and in buffer on the same cells (Figure 5b). Supporting the data of Figure 4, which depicts the elasticity of a single cell and the average stiffness data for the entire sample, confirms an identical effect of dehydration and the same trend along with aging. This includes the progressive stiffening of the cells and the reduction of the differences between air and liquid at increasing times.

The analogy between roughness and stiffness, where an increase in stiffness is formally (but also logically) coupled to a decrease in roughness, should be emphasized. Indeed, the combination of morphometric and nanomechanical data describes aging as a process during which the biophysical properties vary as cells undergo, on the one hand, an ultra-morphological (membrane) and structural (cytoskeleton) rearrangement, measured by roughness, and, on the other hand, a structural stiffening and dehydration, as measured by nanomechanical data. The internal correlation between these biophysical events is intrinsic and is supported by additional observations. For example, the evidence indicates that, during the phases of prevalent cytoskeleton detachment from the membrane associated with a decrease in roughness, a general stiffening of the cellular structure can be observed [74,75,76]. Furthermore, it is interesting to note that RBC aging intrinsically contains some form of dehydration since, due to ionic imbalances mediated by energy availability and cellular metabolism, senescent cells can lose up to 20–30% of their Hb content, fluids, and membrane surface [12,77]. In this regard, it is remarkable that the trend of roughness measured along the aging and during dehydration gives a coherent interpretation of the two phenomena. This also extends to the observation that during dehydration, young cells lose more water than old cells.

**Figure 6:**
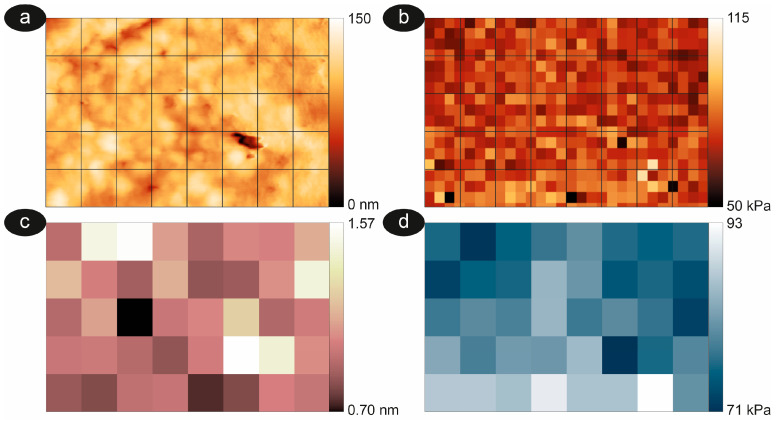
Comparison between a morphological image (panel (**a**), 4 × 2.5 μm^2^), the corresponding stiffness map (panel (**b**)), maps of roughness (panel (**c**)), and Young’s modulus (panel (**d**)) performed on the very same biconcave cell in liquid and reported in a false color scale. For the roughness, the values were calculated on a 500 × 500 nm^2^ size (black squares on panel (**a**)). For elasticity, the average value was calculated, averaging the Young’s moduli from the force curves comprising every square.

Concerning the analogy between roughness and stiffness (or Young’s modulus), in which a decrease in roughness is associated with an increase in stiffness, it is interesting to understand whether these two parameters produce an identical description of the sample. To this end, in Figure 6, we report a comparison between a map of roughness and of mechanical properties, calculated in liquid and on a dimensional scale of 500 × 500 nm^2^. This evaluation highlights that the mapping of the cell roughness and of the cell stiffness is different and cannot be straightforwardly correlated. It must be stressed that an extensive comparison of the data ensured that the differences between these parameters are deep, and do not depend on the size of the area considered, nor on the morphological phenotype chosen.

These results suggest that roughness and nanomechanical properties are distinct parameters and do not measure the same physical characteristics of the RBC; thus, a combined analysis represents a non-redundant and complementary approach to better describe the erythrocytes’ properties. Yet, a deeper discussion of the two parameters and of their relationships is in order.

Nanomechanical properties have been widely discussed and modelled, and it is known that their direct measurement provides information on the elastic or viscoelastic properties of biosystems [78,79]. It is also known the role that these properties play in different physiological (for example, mediating the response to environmental stimuli) and pathological contexts (where they can mediate transitions in cellular behavior in different phases of the development of a disease) [44,45].

On the other hand, the biological significance of roughness is more complex. From a biophysical point of view, previous studies allowed determining the morphological alterations associated with biochemical conditions, such as a lack of energy resources, reduced power, oxidation states, and others. The roughness values in RBCs typically decrease in time-dependent studies such as the aging process, or when comparing healthy and pathological samples (for example, with cytoskeletal defects).

More specifically, the decrease in roughness correlates quite well with the trend of intracellular ATP, also in terms of reversibility of morphological alterations following cell revitalization treatments [17,21]. This is functionally important because it has been shown that ATP-dependent phosphorylation regulates the adhesion of the cytoskeleton to the plasma membrane [73,80]. This creates a correlation between the mechanical and morphological properties of red blood cells that, in fact, can be measured with remarkable efficacy by the membrane roughness. No less important is the affinity of the cytoskeleton for the membrane, which, through the function of the band 3 protein, is also involved in the oxygen-dependent regulation of glycolysis [81]. Therefore, it is clear that the membrane roughness is not just a simple morphological parameter, as it is quantitatively sensitive to cellular pathways and can provide an indirect evaluation of cellular metabolic activity.

From an experimental point of view, the calculation of the roughness and the operating protocol are simple; however, the AFM measurement is (moderately) perturbative since it is associated with a local pressure exerted on the membrane by the AFM sensor. Thus, it is expected that the mechanical properties of the membrane can play a role in terms of reversible deformation associated with the measurements. On the other hand, on the nanometric scale, as a biological membrane is only locally supported by the cytoskeleton, the measurement of roughness is mediated by a highly discontinuous function. Yet, it is precisely through this mechanism of compliance mediated by the different mechanical support that the roughness efficiently translates the weakening of erythrocyte structure into a reduction of roughness values. This occurs in aging, as well as in many other experimental conditions [22]. In light of these considerations, we must think that roughness, at least in the case of biosystems, contains a morphological component, but also retains the fingerprint of the local mechanical structure of the cell. Therefore, it is not surprising that an attempt to associate it with a parameter such as the local stiffness (or analogous, single, nanomechanical property) is partly frustrating.

However, the nature of roughness measurement, which depends on spatial sampling, could suggest a method to further analyze the roughness. Indeed, whatever the influence of local mechanics on the measurement is, it certainly could not alter the topological nature of the surface under study. This is particularly interesting if we consider the biological structure of the erythrocyte membrane which, as a surface, has a certain characteristic of self-similarity. For surfaces with self-similar properties, the trend of the roughness (*R*) can be described according to the following law:

(2) $$R=k\times L^{\alpha }$$

where *L* is the size of the sampling area (a square, in our case), *k* is a normalization factor and *α* is a coefficient with values ranging from 0 (if the values of the surface follow a white noise pattern) to 1.

**Figure 7:**
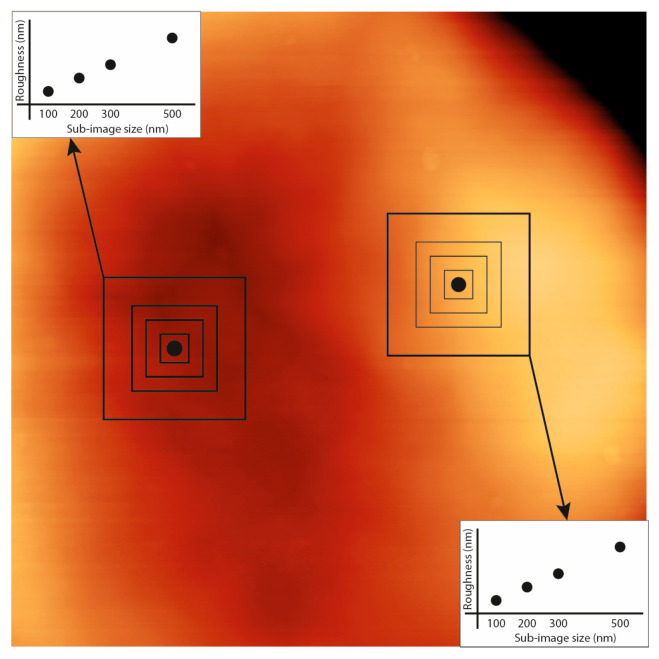
The determination of the topology-dependence of the roughness was calculated by averaging the values over different square sizes (100, 200, 300, and 500 nm^2^) centered on a selected point on the surface, where a given force curve was collected. The plot of the roughness values was then fitted to determine the α value. The overall image is 2.1 × 2.1 μm^2^.

In this framework, the roughness grows with a power law that, at small sizes (those investigated in our analyses), has a linear phase, and that saturates at larger lengths. The exponent α, calculated with this approach, allows condensing the scale geometries of the membrane in a numerical factor, that, by properly evidencing the contribution of the surface topology to the measure of the roughness, would provide an effective comparison with the nanomechanical data. Such a multiscale approach, certainly innovative for the proposed methodology and conceptual development, requires an in-depth evaluation that goes beyond the scope of this study. However, preliminarily, we have developed a methodological analysis, according to a scheme reported in Figure 7, in which the surface sampling method and the calculation of the self-similarity factor α are indicated.

Physically speaking, we expect that an environmental effector that has an impact on biological structures can determine alterations of the topological characteristics and the spatial correlation of the membrane structures. Such alterations, then, would be measurable and analyzable in terms of changes of the surface geometry. In this sense, the analysis of roughness as a function of spatial scale can contain new and still unexplored information useful per se, but also to compare roughness and nanomechanical properties.

It is interesting to note that, in the scientific test case we proposed, the value of α showed a promising sensitivity to the different biophysical conditions to which the samples were exposed.

## 4. Conclusions

In the present paper, we employed two distinct approaches to compare the information obtained in the study of a biosystem: the direct measurement of the local nanomechanical properties of cells and the measurement of the membrane roughness, a statistical morphometric parameter. These two parameters are very different in nature. They require different data collection and point to a distinct description of the cell’s characteristics, and they have never been considered from a common perspective. In this view, describing the methodology to properly analyze and compare the different data is very important, which was the topic of the first part of this study. The study effectively tackled several key technical issues, including how to ensure comparable resolution images in air and liquid, warrant nanometric positioning accuracy when correlating roughness-to-roughness or roughness-to-nanomechanical data, and standardize the collection and processing of force curves.

As a test case, we analyzed the alterations induced by dehydration on erythrocytes by monitoring the change in cell stiffness and the corresponding morphological rearrangement at the nanoscale, obtaining a comprehensive and coherent description of the changes in the cellular properties.

Subsequently, we applied this methodology to the study of human RBCs aging in starvation conditions, a topic that we already investigated and described in previous papers, in which complete biological and biophysical details were detailed in previous papers [20,21,22,27]. We performed correlative measurements combining optical and AFM images, comparing the data in dehydrated and in liquid samples. Coupling the information collected through the membrane roughness and the nanomechanical properties, we were able to describe aging as a process during which several biophysical properties change in an interconnected fashion, as the cells become progressively more rigid and the skeletal support of the membrane degrades. At the same time, we showed evidence of change in the hydration ability of the cells, as young cells lose much more water content during dehydration than senescent cells. This last phenomenon probably reflects an ionic imbalance occurring during aging.

Overall, the data presented in this study, on the one hand, provides a protocol combining conventional and ultra-microscopy on the very same cells, to compare mechanical and morphometric cellular parameters, while, on the other hand, it clarifies that roughness and stiffness do not represent two different measurements of the same physical quantity or of the same cell property. Indeed, besides a common dependency on the mechanical cell response, roughness has an additional non-negligible role for the membrane surface topology, which should be considered. Consequently, a novel approach, coupling the simultaneous measurements of nanomechanical properties and surface roughness, can be recommended as a promising method to provide a more accurate description of the properties of cells and of their alterations during the progress of biological phenomena.

In conclusion, the data presented here, although not in physiological conditions, already allow understanding how the cell properties are interrelated and change during a complex phenomenon such as aging. The construction of this biophysical frame, properly supported by solid statistics on the data, is the necessary base for a future extension of the present methodology to the study of live cells.

## Data Availability

All data presented in these studies are archived in CNR-ISM repository and will be freely available upon reasonable requests.
